# The incretin co-agonist tirzepatide requires GIPR for hormone secretion from human islets

**DOI:** 10.1038/s42255-023-00811-0

**Published:** 2023-06-05

**Authors:** Kimberley El, Jonathan D. Douros, Francis S. Willard, Aaron Novikoff, Ashot Sargsyan, Diego Perez-Tilve, David B. Wainscott, Bin Yang, Alex Chen, Donald Wothe, Callum Coupland, Mattias H. Tschöp, Brian Finan, David A. D’Alessio, Kyle W. Sloop, Timo D. Müller, Jonathan E. Campbell

**Affiliations:** 1 Duke Molecular Physiology Institute, Durham, NC USA; 2 Novo Nordisk Research Center, Indianapolis, IN USA; 3 Lilly Research Laboratories, Eli Lilly and Company, Indianapolis, IN USA; 4 Institute for Diabetes and Obesity, Helmholtz Zentrum München, Neuherberg, Germany; 5 German Center for Diabetes Research (DZD), Neuherberg, Germany; 6 Department of Internal Medicine, University of Cincinnati College of Medicine, Cincinnati, OH USA; 7 Helmholtz Zentum München, Neuherberg, Germany; 8 Technische Universität München, München, Germany; 9 Division of Endocrinology, Department of Medicine, Duke University, Durham, NC USA; 10 Department of Pharmacology and Cancer Biology, Duke University, Durham, NC USA

**Keywords:** Pharmacokinetics, Type 2 diabetes, Obesity, Metabolic diseases

## Abstract

The incretins glucose-dependent insulinotropic polypeptide (GIP) and glucagon-like peptide 1 (GLP-1) mediate insulin responses that are proportionate to nutrient intake to facilitate glucose tolerance^1^. The GLP-1 receptor (GLP-1R) is an established drug target for the treatment of diabetes and obesity^2^, whereas the therapeutic potential of the GIP receptor (GIPR) is a subject of debate. Tirzepatide is an agonist at both the GIPR and GLP-1R and is a highly effective treatment for type 2 diabetes and obesity^3,4^. However, although tirzepatide activates GIPR in cell lines and mouse models, it is not clear whether or how dual agonism contributes to its therapeutic benefit. Islet beta cells express both the GLP-1R and the GIPR, and insulin secretion is an established mechanism by which incretin agonists improve glycemic control^5^. Here, we show that in mouse islets, tirzepatide stimulates insulin secretion predominantly through the GLP-1R, owing to reduced potency at the mouse GIPR. However, in human islets, antagonizing GIPR activity consistently decreases the insulin response to tirzepatide. Moreover, tirzepatide enhances glucagon secretion and somatostatin secretion in human islets. These data demonstrate that tirzepatide stimulates islet hormone secretion from human islets through both incretin receptors.

## Abstract (web summary)

This study shows that the insulinotropic actions of the dual GIPR and GLP-1R agonist, tirzepatide, depend on GIPR, and that tirzepatide has weaker activity at the mouse GIPR than murine GIP.

## Main

The incretin axis is responsible for most postprandial insulin secretion in healthy humans, and loss of the incretin effect contributes to impaired glycemic control in people with type 2 diabetes^6^. Based on these characteristics, the incretin axis continues to be an attractive target for drug development, and GLP-1R agonists (GLP-1RA) have emerged as potent and effective treatments to reduce blood glucose and body weight^7^. Continued evolution of this drug class has seen the development of single peptides that activate multiple receptors, with incretin peptide sequences engineered to activate additional G-protein-coupled receptors (GPCRs)^8^. An early monomeric dual receptor agonist targeted both GLP-1R and GIPR, and the synergism between the two receptor systems was initially reported 10 years ago. In mouse models, dual agonism had superior efficacy for weight loss and glucose control compared to a GLP-1RA alone^9^, with additive effects of the GIPR proposed to act through signaling in beta cells^10^, alpha cells^11^, the CNS (central nervous system)^12,13^ and adipocytes^14^. Despite promising preclinical studies, a 12-week clinical trial using an iteration of this initial dual-incretin agonist failed to demonstrate superiority relative to a GLP-1R monoagonist^15^, raising questions about multi-receptor strategies in humans.

Tirzepatide is an agonist for both incretin receptors, engineered from the human GIP (hGIP) peptide sequence. It has an average half-life of approximately 5 days, enabling once-weekly dosing^5^. Tirzepatide is an imbalanced agonist, engaging the GIPR to a greater degree than the GLP-1R in cultured cell systems. Moreover, it has an in vitro pharmacological profile that mimics the signaling of native GIP at the GIPR, but it is biased at the GLP-1R to favor cyclic AMP (cAMP) generation over β-arrestin recruitment^16^. In clinical trials, treatment with tirzepatide produced superior weight loss and glycemic control compared to GLP-1RAs^3,17^, suggesting that agonism at both the GIPR and GLP-1R is beneficial in humans with type 2 diabetes. However, despite strong evidence that tirzepatide engages the GIPR in competition-binding assays, cell-based experiments using transfected receptors and studies of transgenic mice, there is little functional evidence that tirzepatide directly activates the GIPR in humans as part of its substantial pharmacological effect.

Here, we report the results of experiments with tirzepatide in primary islets, an experimental approach uniquely suited to assess whether tirzepatide directly activates the GIPR in humans. Beta cell incretin receptor activity drives insulin secretion, an essential component of the antidiabetic response to either GLP-1R or GIPR agonists^10,18^. Additionally, the beta cell is one of only a few cell types that express both incretin receptors, providing a model to test the relative importance of GIPR versus GLP-1R signaling. The GLP-1 sequence is conserved across rodent and human species, whereas the GIP sequence differs between species. Tirzepatide is engineered from the hGIP sequence^5^. Importantly, hGIP has reduced potency at the mGIPR^19^, and it has been suggested that tirzepatide also has reduced potency at the mGIPR^20^. Therefore, our initial investigation set out to provide a comprehensive analysis of the potency of tirzepatide at the mGIPR to identify potential limitations of using mouse models to study the actions of tirzepatide. We assessed target engagement of mGIP, hGIP and tirzepatide at the mGIPR with four complementary approaches: (1) ligand binding assays; (2) Gα_S_ recruitment; (3) G-protein activation; and (4) cAMP generation (Table 1). Overall, the affinity–potency profile of tirzepatide was 3–60-fold weaker relative to mGIP at the mGIPR, with similar or slightly reduced potency compared to GLP-1 at the mGLP-1R (Extended Data Table 1). Previous measures of tirzepatide activation of human incretin receptors demonstrated increased potency at hGIPR relative to hGLP-1R^16^. Based on these early studies, it was concluded that tirzepatide acts on the hGIPR similarly to native hGIP but engages the hGLP-1R with parameters that differ from native GLP-1. However, this profile appears to differ for tirzepatide interactions with mouse incretin receptors; for example, tirzepatide and GLP-1 behave similarly at the mGLP-1R, whereas tirzepatide is less potent at the mGIPR than mGIP. This suggests that the imbalanced activity of tirzepatide may actually favor GLP-1R signaling in murine beta cells and suggests caution when using the compound in experiments with mouse models.

**Table 1:** Phamacokinetics of tirzepatide, mouse GIP and human GIP at the mouse GIP receptor

	[^125^I]GIP(1-42)OH binding	G_s_ recruitment		[^35^S]GTPγS binding		cAMP	
Peptide	Ki (nM)	EC_50_ (nM)	E_max_ (%)	EC_50_ (nM)	E_max_ (%)	EC_50_ (pM)	E_max_ (%)
mGIP	0.764 (0.182, 3)	61.4 (9.8, 9)	100 (2.46, 9)	0.181 (2.9, 6)	98.9 (2.9, 6)	6.04 (2.0, 3)	103 (3.9, 3)
hGIP	n/a	152.6 (19.0, 9)*	101 (2.45, 9)	0.735 (0.068, 13)	96.7 (1.3, 13)	46.0 (16.0, 3)	102 (4.7, 3)
TZP	24.3 (1.7, 3)*	153.1 (18.6, 9)*	72.6 (1.73, 9)*	5.35 (0.901, 7)*^,^ **	88.9 (1.4, 7)*^,^ **	363 (68.0, 3)*^,^ **	103 (3.5, 3)

Values are expressed as mean (s.e.m., n). A Student’s *t*-test was used to compare differences with GIP binding, and a one-way ANOVA with Tukey’s post-hoc test was used for all other comparisons.

*P < 0.05 versus mGIP

**P < 0.05 versus hGIP.

Source data

**Fig. 1:**
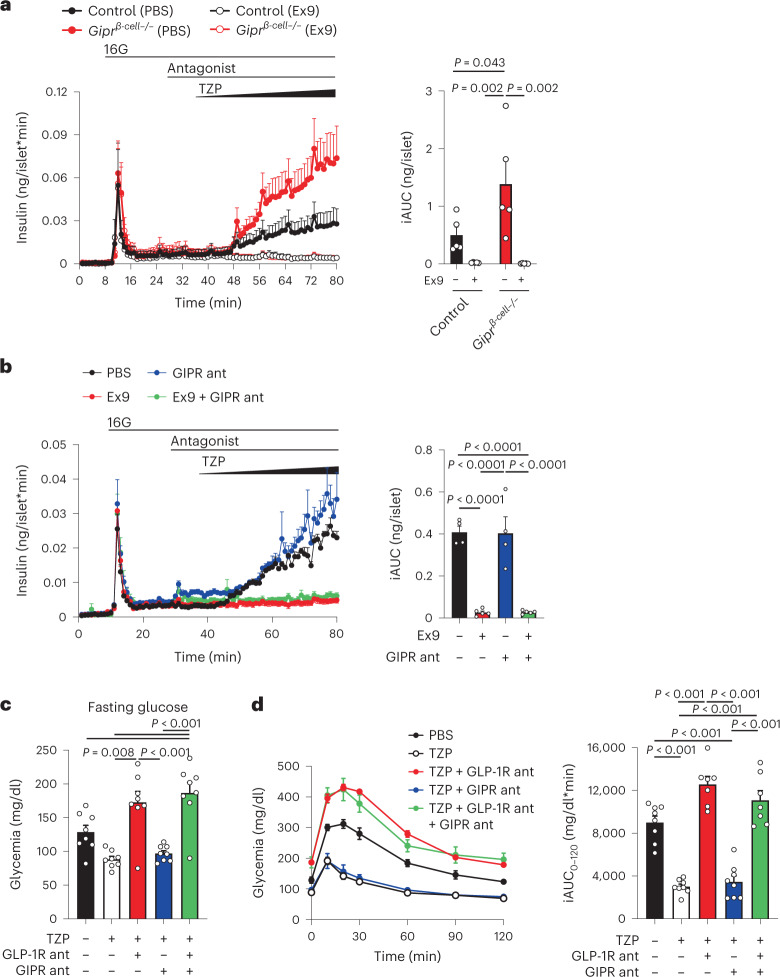
Tirzepatide stimulates insulin secretion in mice predominantly through the GLP-1R. **a**, Mouse islets from control mice and mice with beta-cell-specific deletion of the *Gipr* (*Gipr*^*-β-cell-/-*^) were perifused with ramping concentrations of tirzepatide (TZP) (0–100 nM) with or without Ex9 at a 1 μM concentration beginning at minute 28. The iAUC was calculated for TZP using the value at minute 42 as the baseline. *n* = 5 for all groups. **b**, Mouse islets were perifused with increasing concentrations of TZP (0–100 nM) in the presence of Ex9, a GIPR antagonist (GIPR ant) or a combination of both. All antagonists were used at 1 μM concentrations starting at minute 28. The iAUC was calculated for TZP using the value at minute 42 as the baseline. PBS and GIPR ant, *n* = 4; Ex9 and Ex9 + GIPR ant, *n* = 5. **c**, Glycemia after a 5 h fast, immediately before the administration of glucose. Antagonists were administered 2 h before and TZP was administered 1 h before. *n* = 8 for all groups. **d**, Glycemia during the IPGTT. The iAUC was calculated using the fasting glycemia value. PBS, TZP and TZP + GIPR ant, *n* = 8; TZP + GLP-1R ant and TZP + GLP-1R/GIPR ant, *n* = 7. All values are mean ± s.e.m. Statistical tests were two-way ANOVA with Tukey’s post-hoc test (**a**) and one-way ANOVA with Tukey’s post-hoc test (**b**–**d**). Source data

**Extended Data Fig. 1:**
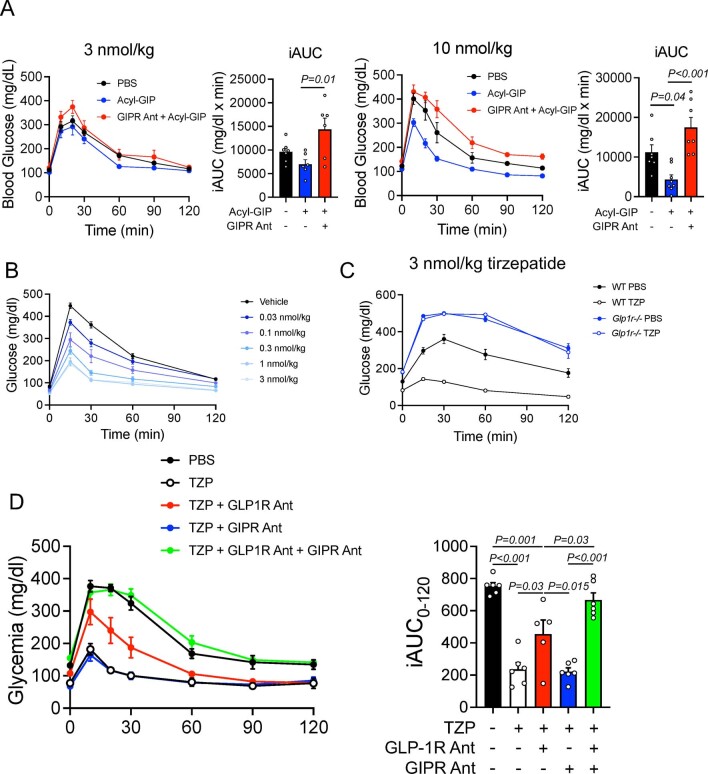
Tirzepatide activity in mice. A) Intraperitoneal glucose tolerance test (IPGTT) in wild-type (WT) mice. Mice were pretreated 2 hours prior to glucose with either PBS or a GIPR antagonist, 1 hour prior to glucose with either PBS or acyl-GIP (3 or 10 nmol/kg dose). Glucose was given after a 5 hour fast at 1.5 mg/kg. The integrated area under the curve (iAUC) was calculated using the glycemia measure immediately before PBS/GIPR antagonist administration. 3 nmol/kg dose: PBS/PBS, n = 7; PBS/Acyl-GIP, n = 6, GIPR Antag/Acyl-GIP, n = 6. 10 nmol/kg dose: PBS/PBS, n = 7; PBS/Acyl-GIP, n = 8, GIPR Antag/Acyl-GIP, n = 7. B) A dose-response curve for tirzepatide during an IPGTT in WT mice. (n = 5/group) B) Tirzepatide during an IPGTT in WT and Glp1r knockout mice. (n = 8/group) C) 30 nmol/kg tirzepatide in WT mice treated with an antagonist for the GLP-1R (Jant-4), GIPR, or both. N = 8/group. All values are mean + /- SEM. The following statistical tests were used: A, B, and D) one-way ANOVA with Tukey’s posthoc test, C) two-way ANOVA with Tukey’s posthoc test. Source data

To address the functional importance of tirzepatide at each incretin receptor, we investigated the effect of loss-of-function approaches on insulin secretion in mice. We first used islets isolated from mice with selective deletion of the *Gipr* in beta cells in combination with the GLP-1R antagonist exendin(9-39) (Ex9). Tirzepatide stimulated insulin secretion in a concentration-dependent manner (0–100 nM) in control islets (Fig. 1a). However, compared to control islets, beta cell *Gipr* knockout islets secreted more insulin in response to tirzepatide (Fig. 1a), whereas Ex9 completely blocked insulin secretion in response to tirzepatide in both control and knockout islets (Fig. 1a). We reasoned that the enhanced response in knockout islets was attributed to a compensatory enhancement in GLP-1R signaling that has been previously described in various GIPR knockout models^10,21,22^. To circumvent this issue, we next used acute pharmacological antagonism of the incretin receptors in mouse islets. We applied a recently validated long-acting GIPR antagonist^23^, which prevented glucose lowering in response to an acylated GIPR agonist in wild-type mice (Extended Data Fig. 1a). Antagonism of the GLP-1R with Ex9 prevented insulin secretion in response to tirzepatide, whereas the presence of a GIPR antagonist had no effect on tirzepatide-stimulated insulin secretion, alone or in combination with Ex9 (Fig. 1b). These findings suggest that tirzepatide works predominantly through the GLP-1R to stimulate insulin secretion in mouse islets. To determine the consequences of these findings on glucose tolerance, we pretreated mice with acylated antagonists of GLP-1R^24^ or GIPR, alone or in combination, followed by tirzepatide and an intraperitoneal glucose tolerance test (IPGTT) (Fig. 1b). We used 3 nmol kg^–1^ of tirzepatide, identified as a maximal dose for glucose lowering in wild-type mice (Extended Data Fig. 1b), to provide an opportunity for activity at both incretin receptors. Before glucose administration, tirzepatide reduced fasting glycemia, which was prevented by antagonism of the GLP-1R but not the GIPR (Fig. 1c). Tirzepatide robustly lowered glycemia during the IPGTT, an effect that was completely blocked by GLP-1R antagonism (Fig. 1d) or when the experiments were conducted in *Glp1r*-knockout mice (Extended Data Fig. 1c). In comparison, antagonism of the GIPR did not alter the actions of 3 nmol kg^–1^ of tirzepatide to reduce glycemia or influence the effect of the GLP-1R antagonist on glucose tolerance (Fig. 1d). It has been demonstrated that higher doses of tirzepatide show activity at the GIPR^5^, prompting us to repeat these experiments using 30 nmol kg^–1^ of tirzepatide. We found that GLP-1R antagonism only partially blocked the glucose-lowering effects of tirzepatide (Extended Data Fig. 1c). Moreover, whereas GIPR antagonism alone failed to prevent the effects of this higher dose of tirzepatide, a combined effect of both antagonists was seen, preventing glucose lowering in response to tirzepatide. These data agree with previous results that show that tirzepatide can engage the mGIPR but requires high doses to do so in mice.

**Fig. 2:**
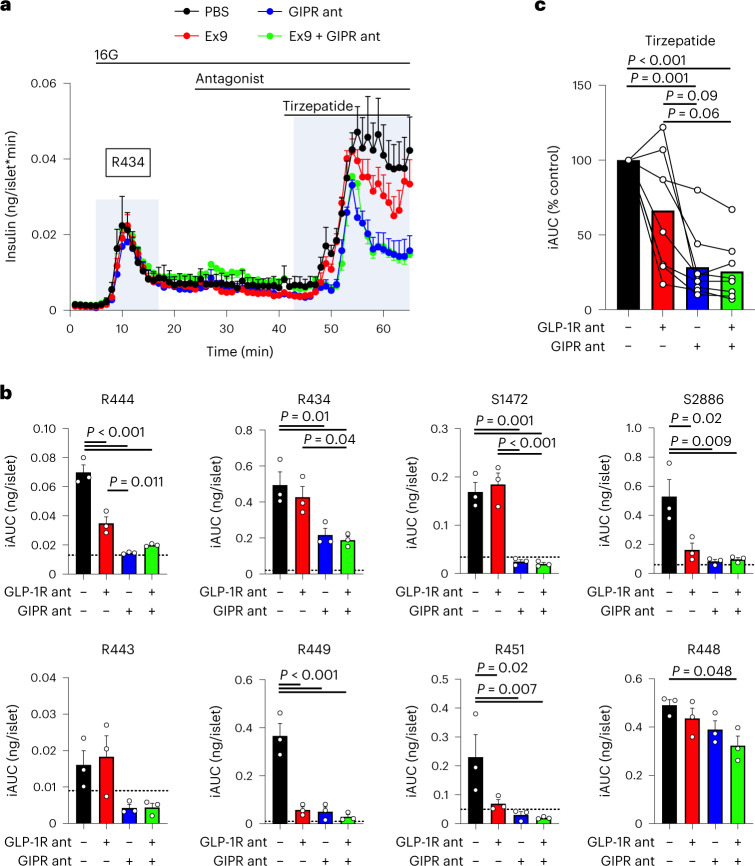
Tirzepatide stimulates insulin secretion through both the GLP-1R and GIPR in human islets. **a**, Islet perifusion from one set of human islets. Insulin secretion was measured in response to 30 nM tirzepatide in the presence of Ex9 (1 μM), a GIPR antagonist (GIPR ant) or both. *n* = 3 per group. **b**, iAUC values for insulin secretion in response to tirzepatide in eight individual sets of human islets. The dashed line indicates the level of glucose-stimulated insulin secretion before tirzepatide stimulation. *n* = 3 per group. **c**, Summary data of all eight experiments in human islets. Each individual experiment was averaged to produce a single data point for each condition and expressed as a relative value to control conditions. Individual experiments have connecting lines. *n* = 8. All values are mean ± s.e.m. The statistical test was one-way ANOVA with Tukey’s post-hoc test (**b**,**c**). Source data

**Extended Data Fig. 2:**
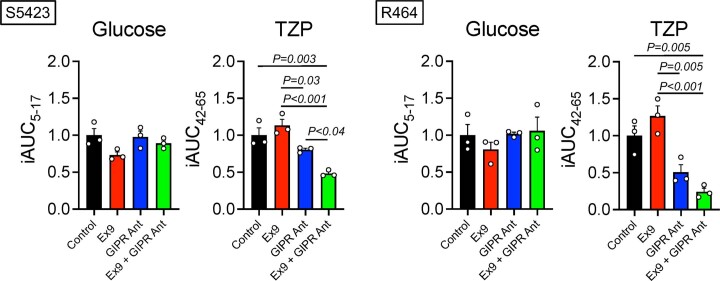
Tirzepatide stimulated insulin secretion in human islets. Two independent donor sets of human islets were stimulated with 30 nM tirzepatide (TZP) in the presence of 1μM concentrations of exendin(9-39) (Ex9), hGIP(3-30) (GIPR Ant) or the combination. The integrated area under the curve (iAUC) was calculated for both glucose and tirzepatide stimulated insulin secretion using the value of the first time point as the baseline. All values are mean + /- SEM, n = 3/group. A one-way ANOVA with Tukey’s posthoc test was used. Source data

Tirzepatide favors the GLP-1R over the GIPR at mouse incretin receptors, whereas the opposite has been reported for the relative potency in human incretin receptors, for which tirzepatide activity is tilted towards the GIPR^5,16^. Although our mouse studies suggest that tirzepatide stimulates insulin secretion predominantly through the GLP-1R, the species difference in receptor pharmacology may limit extension of this interpretation to humans^25,26^. Therefore, we next determined which incretin receptor tirzepatide uses to stimulate insulin secretion in isolated islets from human donors. We used 30 nM of tirzepatide to align with concentrations previously published^5^. In an experiment from a single representative donor, antagonizing the GLP-1R failed to reduce tirzepatide-stimulated insulin secretion, whereas antagonizing the GIPR reduced insulin secretion by ~55% (Fig. 2a). Owing to donor-to-donor variability associated with human samples, we repeated this protocol in additional sets of islets from humans that spanned a range of BMI, age and HbA1C%, and included donors of both sexes (Extended Data Table 2). In these studies, the effect of GLP-1R antagonism varied among islet preparations, failing to reduce tirzepatide-stimulated insulin secretion in nearly half of the experiments (Fig. 2b), whereas antagonism of the GIPR consistently decreased tirzepatide-stimulated insulin secretion across all donor sets (Fig. 2b). When all the islet preparations are averaged and expressed as a per cent reduction relative to control conditions, GLP-1R antagonism alone did not reduce insulin secretion significantly, probably due to the high degree of variability amongst the various donors (Fig. 2c). However, GIPR antagonism alone decreased tirzepatide-stimulated insulin secretion relative to both PBS and GLP-1R antagonism. Finally, the addition of the two antagonists did not produce an effect that was different from GIPR antagonism alone. A similar outcome was seen when using hGIP(3-30) (Extended Data Fig. 2), a different GIPR antagonist that has been previously characterized^27^. We conclude from these studies that the insulinotropic effects of tirzepatide are mediated by both receptors, but that the contribution of each receptor varied from donor to donor; there was no correlation with available donor characteristics or the rate of glucose-stimulated insulin secretion. Furthermore, antagonizing the GIPR reduced tirzepatide-stimulated insulin secretion in each islet set, demonstrating that activity at the GIPR is necessary for tirzepatide to stimulate insulin secretion in isolated human islets.

**Fig. 3:**
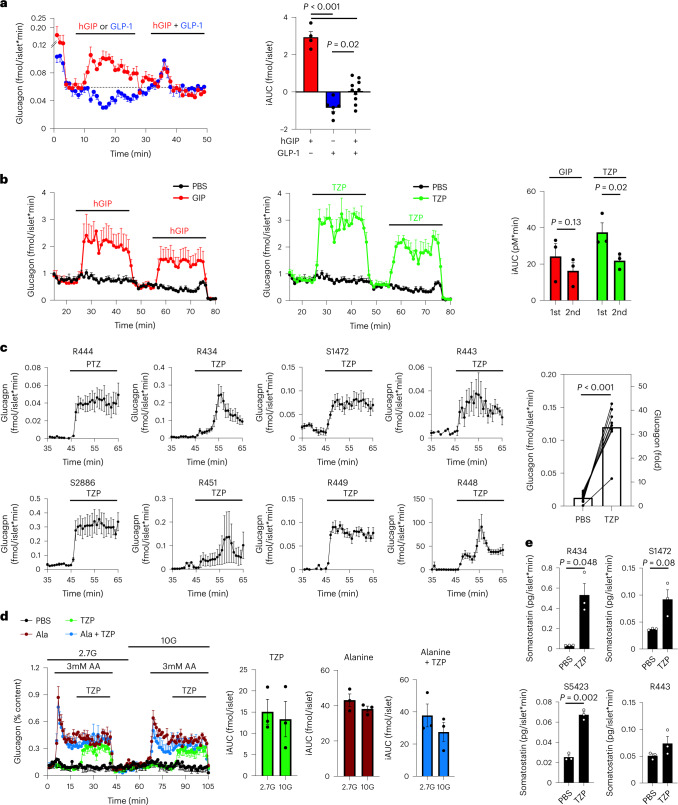
Tirzepatide stimulates glucagon secretion in human islets. **a**, Glucagon secretion from human islets stimulated with either 30 nM hGIP or GLP-1 individually (minutes 8–24) or together (minutes 32–50) under 16 mM glucose conditions. The iAUC was calculated for the individual effects of the peptides (minutes 8–24) and the combined effects (minutes 32–50) using the value of the first time point as the baseline. GIP, *n* = 4; GLP-1, *n* = 6; GIP + GLP-1, *n* = 10. **b**, Glucagon secretion from human islets treated with 30 nM of either hGIP or tirzepatide (TZP) under 16 mM glucose conditions. The iAUC was calculated using minutes 24 and 54 as the baseline values for the first and second stimulation, respectively. *n* = 3 for all groups. **c**, Glucagon secretion in response to 30 nM TZP in eight individual donor sets of human islets under 16 mM glucose conditions. The summary of these experiments is shown as the of the average values from minutes 55–65. The fold induction on the right *y* axis was calculated using the baseline glucagon values from minutes 35–45. *n* = 3 for each donor, *n* = 8 for summary data. **d**, Glucagon secretion in human islets (donor R464) stimulated with TZP (30 nM) at either 2.7 mM glucose (2.7 G) or 10 mM glucose (10 G), with or without 3 mM alanine. *n* = 3. **e**, Somatostatin concentrations from pooled samples taken from baseline samples or during TZP stimulation. *n* = 3. All values are mean ± s.e.m. Statistical tests were one-way ANOVA with Tukey’s post-hoc test (**a**), two-way ANOVA with Sidak post-hoc test (**b**), paired *t*-test (**c**,**d**) and unpaired *t*-test (**e**). Source data

**Extended Data Fig. 3:**
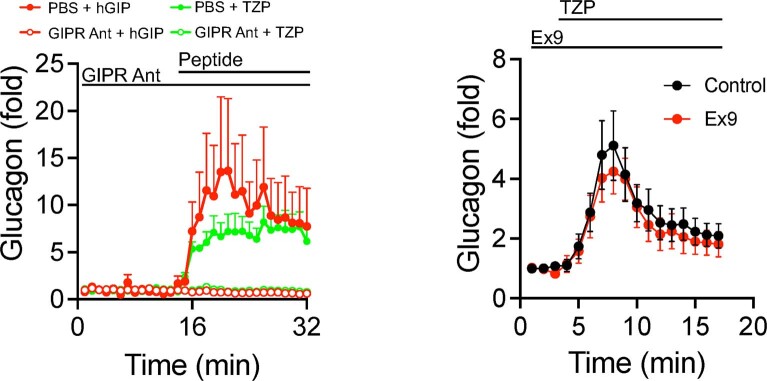
GIPR but not GLP-1R is needed for tirzepatide-stimulated glucagon secretion. Human islets were stimulated with either tirzepatide or hGIP in the presence of the GIPR antagonist (GIPR Ant – left panel) or exendin(9-39) (Ex9 – right). Tirzepatide was used at 30 nM and the antagonists were used at 1μM concentrations. All values are mean + /- SEM, n = 3/group for GIPR antagonist and 6/group for Ex9. A two-way ANOVA with Tukey’s posthoc test was used. Source data

**Extended Data Fig. 4:**
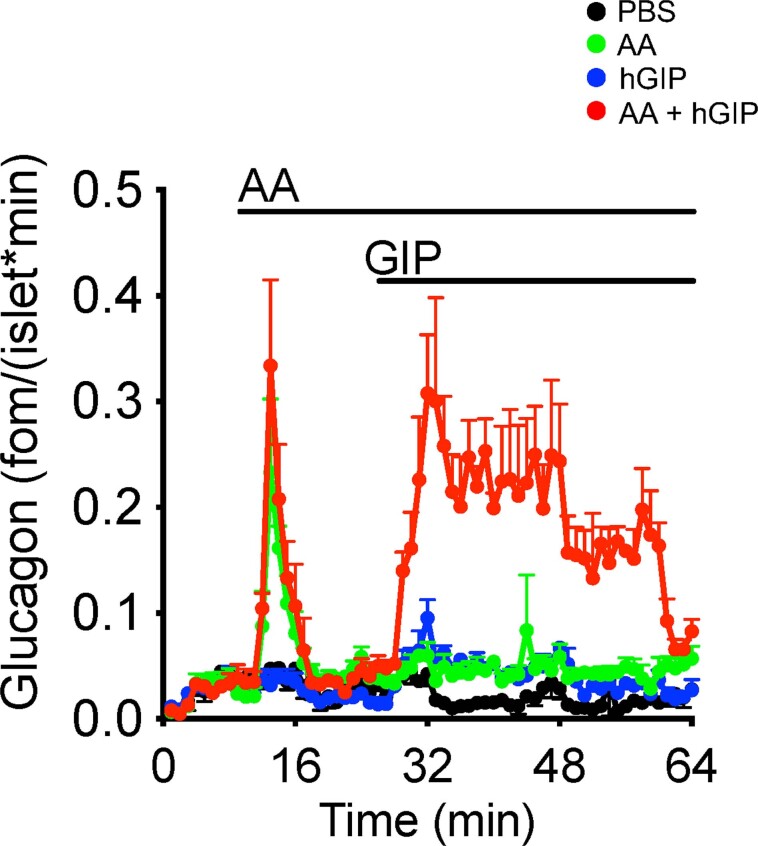
GIP and amino acids synergistically increase glucose secretion in human islets. Human islets were treated with 30 nM hGIP or 3 mM amino acids (combination of glutamine, arginine, alanine, and leucine), individually or in combination. All values are mean ± SEM, n = 3/group. A two-way ANOVA with Tukey’s posthoc test was used. Source data

Next, we took advantage of the known actions of islet incretin receptors on glucagon secretion from alpha cells as an orthogonal approach to assess the relative contribution of tirzepatide at each receptor. GLP-1R agonism has well-established actions to reduce glucagon secretion in both isolated human islets^28^ and in vivo^29^, whereas GIPR agonism increases glucagon secretion in preclinical models^11^ and in humans^30^. Interestingly, the combination of GLP-1 and GIP on glucagon secretion is reported either to offset^31–33^ or to decrease glucagon levels^34^. Studies showing that the combined actions of both incretin receptor agonists decrease glucagon secretion suggest that the inhibitory actions of GLP-1R agonism outweigh the stimulatory actions of GIPR agonism. In isolated human islets, we confirmed that hGIP stimulated glucagon secretion, GLP-1 decreased glucagon secretion and the combination of the two peptides offset each other to produce a rate of glucagon secretion that matched unstimulated levels (Fig. 3a). In addition, we found that tirzepatide produced a similar increase in glucagon secretion compared to equal molar concentrations of hGIP in a single donor set of human islets (Fig. 3b). Interestingly, subsequent stimulation with both hGIP and tirzepatide reduced the effects on glucagon secretion, suggesting a degree of desensitization, although only tirzepatide produced a significant effect (Fig. 3b). Moreover, we were able to demonstrate that antagonism of the GIPR, but not GLP-1R, completely blocked the ability of either hGIP or tirzepatide to stimulate glucagon secretion (Extended Data Fig. 3). Of note, the glucagon secretion across different donor sets of human islets was consistent. In all sets of islets, tirzepatide consistently increased glucagon secretion, although the magnitude of glucagon secretion varied amongst donors (Fig. 3c). This finding demonstrates the robust activity of tirzepatide at the alpha cell GIPR that outweighs inhibitory actions produced by GLP-1R agonism and provides additional evidence that tirzepatide has important activity at the GIPR in human islets. We previously showed that GIPR activity in alpha cells potentiates amino-acid-stimulated glucagon secretion in mouse islets^11^, and here we found the same interaction between hGIP and amino acids in human islets using concentrations of amino acids found in the postprandial state (Extended Data Figure 4)^11^, prompting us to ask whether tirzepatide engagement of the GIPR in human islets also enhances the effect of amino acids on glucagon secretion. Interestingly, we found that the effect of tirzepatide on glucagon secretion was independent of glucose concentration or the presence of amino acids (Fig. 3d), suggesting a divergent mechanism from hGIP in alpha cells. Finally, we found that tirzepatide consistently increased somatostatin secretion (Fig. 3e), suggesting an engagement with ẟ-cells that is consistent with activity at both the GIPR and GLP-1R^35,36^. Together, our work shows that tirzepatide produces an increase in all three major islet hormones, displaying functional activity at both incretin receptors in human islets.

In summary, these results provide several important advances in our understanding of tirzepatide pharmacology. First, the insulinotropic actions of tirzepatide in human islets are dependent upon GIPR activity. This directly addresses the questions of whether tirzepatide is simply a more potent GLP-1R agonist and whether activation of the GIPR contributes to metabolic outcomes. Using insulin secretion as a read-out in primary human islets provides a platform to interrogate this question in a system that is intimately linked to the glycemic benefits of tirzepatide. Although this line of investigation would best be undertaken with in vivo studies in humans with and without diabetes, the extended pharmacokinetics make acute studies with tirzepatide directed at beta cell function challenging to design. Indeed, the idea that the insulinotropic actions of GIP are absent in subjects with T2D^29^ has stalled the development of GIPR agonists as a therapy for hyperglycemia. However, emerging evidence requires this idea to be reconsidered. First, a recent study showed that GIPR antagonism reduces insulin secretion during a meal-tolerance test in subjects with T2D^37,38^. This observation highlights that endogenous GIP is essential even in type 2 diabetes, suggesting promise for pharmacological agents that target the GIPR. Second, effective glucose lowering in subjects with type 2 diabetes for 4 weeks enhances the insulinotropic actions of GIP and GLP-1 (refs. ^39,40^). Therefore, it is possible that the relative activity of tirzepatide at the GLP-1R and GIPR may vary in subjects with and without diabetes as well as with the degree of hyperglycemia. In fact, our data demonstrate that even across islets from nondiabetic donors, the relative contribution of GLP-1R versus GIPR to the insulinotropic actions of tirzepatide varies. This is unsurprising given the observation that beta cell sensitivity to GLP-1 in healthy subjects varies up to tenfold^41^. Hence, one potential explanation for the increased efficacy of tirzepatide on glycemic control relative to the monoagonist GLP-1RA is that targeting both incretin receptors maintains some insulinotropic activity even in subjects who are relatively insensitive to GLP-1. Testing these hypotheses merits future effort, as does reconsideration of the previous literature that has shown positive attributes of GIPR agonism for beta cell function^10,42,43^.

A second important observation is that tirzepatide has weaker activity relative to mGIP at the mGIPR. In our studies, antagonizing the GIPR did not affect tirzepatide-stimulated insulin secretion or glycemic control during an IPGTT at doses of 3 nmol kg^–1^, which we identified as maximal for glucose lowering in mice. However, in keeping with our data on receptor pharmacology, increasing the dose to 30 nmol kg^–1^, tenfold higher than maximal doses from our studies, shows that tirzepatide does have insulinotropic activity in *Glp1r*^*-/-*^ mice or in the presence of a GLP-1R antagonist^5^, demonstrating that tirzepatide can engage the GIPR in mouse beta cells at sufficiently high concentrations. Moreover, tirzepatide enhances insulin sensitivity in *Glp1r*^*-/-*^ mice in a manner that is phenocopied by GIPR monoagonism^14^, supporting the activity of tirzepatide at the mGIPR. Therefore, it is not a question of whether tirzepatide can activate the mGIPR, but rather what doses of tirzepatide are required to do so. One important consideration is that the expression patterns of the incretin receptors may determine the dose–response relationship to tirzepatide. In beta cells, which express both incretin receptors, tirzepatide is more potent at the GLP-1R, limiting the activity at the GIPR with lower doses. Higher doses of tirzepatide may allow for engagement of the mGIPR, but it remains unclear whether these high doses alter the effect of the ratio of GLP-1R:GIPR activity, potentially confounding the interpretation of results. Conversely, it may be that cell types that express only one receptor (such as adipose tissue, which only makes GIPR) or certain neuronal populations are less affected by higher doses of tirzepatide. Overall, our results in mice highlight the limitations of using mouse models to study tirzepatide, requiring careful experimental design and interpretation of data in this species.

A final important conclusion from these studies is the robust increase in glucagon secretion induced by tirzepatide. This finding fits with the body of literature that demonstrates that GIPR activity in islets stimulates glucagon secretion^11^. Our finding that tirzepatide increases glucagon secretion in isolated islets not only demonstrates meaningful activity at the GIPR but also provides evidence that tirzepatide can overcome the activity at the GLP-1R on glucagon secretion in human islets. Our studies directly assess the effect of tirzepatide on glucagon secretion, but clinical trials with tirzepatide have reported reductions in both fasting glucagon and the glucagon response from a mixed-meal challenge^17^. Although these data appear to conflict with our observation that tirzepatide stimulates alpha cell activity, it is important to note that the metabolic profile of the subjects in the tirzepatide arm improved dramatically during the 28-week period, complicating interpretations. Indeed, increased metabolic stress elevates both alpha cell and beta cell activity in a compensatory manner, elevating both fasting and stimulated insulin and glucagon levels. Conversely, reductions in body weight and improvements in glycemic control will decrease the compensatory activity of both alpha cells and beta cells, reducing the levels of insulin and glucagon. For example, subjects treated with tirzepatide also showed a reduction in fasting and stimulated insulin levels, data that have not led to the conclusion that tirzepatide inhibits insulin secretion. The implications of tirzepatide-stimulated glucagon secretion warrant further investigation but do suggest new hypotheses centered on the potential metabolic benefits of glucagon agonism. This is particularly interesting as next-generation multi-receptor agonists that incorporate glucagon receptor agonism have shown tremendous promise for weight loss and glycemic control, including triagonists for the GLP-1R, GIPR and GCGR^44,45^.

Our studies in isolated human islets clearly demonstrate a robust action of tirzepatide at the GIPR, both in beta cells and alpha cells. It is important to note that although the human islets we used came from donors with a broad range of metabolic characteristics, we did not have the opportunity to include islets from donors with type 2 diabetes. Furthermore, isolated islets provide a closed system that does not incorporate the full range of regulation present in systemic physiology and may not accurately recapitulate additional details such as blood flow rate or free peptide concentrations, highlighting some of the limitations of this model. Therefore, it is important to extend these studies to human subjects using potent inhibitors of the incretin receptors. However, the data presented here clearly demonstrate that in isolated human islets, tirzepatide requires the GIPR to stimulate both insulin and glucagon secretion.

## Methods

### Ligand-induced BRET assays for G_s_ recruitment

HEK293T cells were maintained at 37 °C in 5% CO_2_ and cultured in DMEM (cat. no. 11995073; Life Technologies) supplemented with 10% heat-inactivated fetal bovine serum (FBS, cat. no. 10500064; Life Technologies), 100 IU ml^–1^ of penicillin, and 100 μg ml^–1^ of streptomycin solution (penicillin-streptomycin, cat. no. P4333; Sigma–Aldrich). While still in log phase, HEK293T cells (700,000 per well) were seeded into 6-well plates (cat. no. 10234832; Fisher Scientific GmbH) in DMEM (10% FBS, 1% penicillin-streptomycin). Twenty-four hours after reaching 70% confluency, transient transfections were performed using Lipofectamine 2000 (cat. no. 11668019; Invitrogen) according to the manufacturer’s protocol. Twenty-four hours following transfection, HEK293T cells were washed with PBS and resuspended in FluoroBrite phenol red-free complete media (cat. no. A1896701; Life Technologies) containing 5% FBS and 2 mM of L-glutamine (cat. no. 25030081; Gibco). Then, 100,000 cells per well were plated into poly-D-lysine-coated (cat. no. P6403; Sigma–Aldrich) 96-well white polystyrene LumiNunc plates (cat. no. 10072151; Fisher Scientific). After 24 h, the media was replaced with HBSS (cat. no. 14025092; Gibco) containing 10 μM of coelenterazine-h (cat. no. S2011; Promega) or 1:500 dilution of NanoGlo (cat. no. N1110; Promega). BRET measurements were taken every 1 min using a PHERAstar FS multi-mode microplate reader. Baseline measurements were taken after an initial 5 min incubation with coelenterazine-h or NanoGlo-containing HBSS, after which cells were treated with either a vehicle (PBS) or the respective ligands. Ligand-specific ratiometric BRET signals were normalized to the vehicle, producing the ‘ligand-induced BRET ratio’^46^, followed by additional normalization to well-specific baseline readings. Ligand-induced measurements on the temporal scale are represented as the subsequent measurement after time point zero. Positive or negative incremental area under the curves (+iAUC, –iAUC) were calculated where noted. Concentration–response curves were generated by three-parameter logistic fitting in Prism 9.0.

### Radioligand binding assays

Membranes from HEK cells expressing cloned human and mouse GLP-1Rs and GIPRs were prepared as previously described^47^. A competitive binding method to quantify the displacement of iodinated GLP-1 and GIP radioligands to their cognate receptors was performed as described in ref. ^16^, with the following modifications. The assay buffer was composed of 2.5 mM MgCl_2_, 1.0 mM CaCl_2_, 0.003% w/v Tween 20, 0.1% w/v bacitracin (USB corporation) in 25 mM HEPES pH 7.4. Approximately 0.05 nM radioligand (Human/Mouse [125I]GLP-1(7-36)NH2 and Human [125I]GIP(1-42)OH; both PerkinElmer >2,200 Ci mmol^–1^, >95% purity) was added to peptide in 100 μl assay buffer (concentration–response curves in DMSO, final concentration of 0.96%) in 96-well plates (cat. no. 3632; Corning). Assay buffer (100 μl) containing membranes that had been preincubated at room temperature with WGA-PVT SPA Beads (PerkinElmer) for 2 h was added. Membrane and bead amounts were as follows: hGLP-1R (0.35 μg protein, 0.125 mg bead), mGLP-1R (0.25 μg protein, 0.125 mg bead), hGIPR (6 μg protein, 0.2 mg bead) and mGIPR (7 μg protein, 0.25 mg bead). Plates were covered with sealing tape (Perkin Elmer), mixed and incubated for 14–16 h at room temperature. Plates were centrifuged at 200×*g* for 5 min, and bound radioactivity was quantified using a scintillation counter (MicroBeta Trilux, PerkinElmer). Total binding was the amount of radioligand bound in the absence of a competitor. Non-specific binding was defined by 100 nM of GLP-1(7-36) or human or mouse GIP(1-42)NH2. *B*_max_ values for radioligands were calculated using homologous competition. IC_50_ values for competitor peptides were calculated using PRISM 7 (GraphPad), and *K*_*i*_ values were calculated using the Cheng–Prusoff correction^48^.

### GTPγs binding and cAMP generation

Methods for these assays have been previously described^16^. In brief, for GTPγs binding, the potency of ligands to stimulate receptor-dependent elevation of the GTPγS-bound Gα_s_ subunit was determined using membrane preparations from low-receptor-density receptor clonal cell lines. Reactions were incubated for 30 min at room temperature in white, clear-bottom microtiter plates, and per cent of the maximal response was calculated using control wells as a reference. Relative EC_50_ values were derived by nonlinear regression analysis using the per cent response versus the concentration of ligand and fitted to a four-parameter logistic equation using GraphPad Prism 7 software. For cAMP assays, kinetic cAMP assays were performed in low-density receptor cell clones transfected with the Glosensor 22 F vector (Promega). Cells were equilibrated for 5–20 min and then 20 μl of 10× ligand was added and a luminescence time course was collected. Ligand (10×) was titrated by manual serial dilution in DMSO followed by step-down into assay buffer.

### Mouse islet perifusion

Details for the general perifusion protocol have been previously described^11^. In brief, islets were individually picked to ensure consistency across chambers with respect to both number and size. A total of 75 islets were loaded into individual chambers and perifused at a constant rate of 200 µl min^–1^ using 2.7 mM glucose Krebs-Ringer-phosphate-HEPES (KRPH) buffer (140 mM NaCl, 4.7 mM KCl, 1.5 mM CaCl_2_, 1 mM NaH_2_PO_4_, 1 mM MgSO_4_, 2 mM NaHCO_3_, 5 mM HEPES and 0.1% FA-free BSA (pH 7.4)) with 100 μl of Bio-Gel P-4 Media (Bio-Rad). The flow rate was based on the manufacturer’s suggestion for this equipment. Changes in glucose concentrations are indicated in the figures and figure legends. The antagonists were used at 1 μM concentrations, previously established to provide full antagonism. Tirzepatide concentration was ramped from 0 to 100 nM. Insulin concentrations are expressed as a function of both rate and islet number (ng ml^–1^ × (1/200 μl ml^–1^) × (1/75 islets))^49^. The iAUC for tirzepatide was calculated from minutes 42–80, using the value at minute 42 as the baseline for each sample, and is expressed as a function of time (ng/(islet × min) × min). Insulin values were measured with a Lumit insulin immunoassay (Promega) using an EnVision plate reader (PerkinElmer).

### Human islet perifusion

Human islets were obtained from both the Alberta Diabetes Institute and the Integrated Islet Distribution Program. All islets were obtained from consenting donors. Details for the general perifusion protocol have been previously described^21^ and are similar to the mouse perifusion protocol. For the insulin secretion experiments (Fig. 2), the iAUC for tirzepatide was calculated from minutes 42–65, using the value at minute 42 as the baseline for each sample. Each set of human islets consisted of *n* = 3 for each condition. These were averaged to generate a single data point (Fig. 2c). Insulin values were measured with a Lumit insulin immunoassay (Promega) using an EnVision plate reader (PerkinElmer). For the glucagon secretion experiments (Fig. 3), the average glucagon values were calculated during control (minutes 35–45) and stimulated (minutes 55–65) conditions. All peptides were used at 30 nM concentrations based on previous work^16^. Amino acids were used at concentrations of 3 mM based on previous work^11^. Glucagon was measured with a Lumit Glucagon Immunoassay Kit (cat. no. CS3037A02; Promega) using an EnVision plate reader (PerkinElmer). Somatostatin was measured with an ELISA assay (cat. no. FEK-060-14; Phoenix Pharmaceuticals). All data are expressed as the measured concentration relative to both flow rate and islet number (insulin, ng ml^–1^ × (1/200 µl ml^–1^) × (1/75 islets); glucagon, pM × (1/200 µl ml^–1^) × (1/75 islets); somatostatin, pg ml^–1^ × (1/200 µl ml^–1^) × (1/75 islets))^49^. Human pancreatic islets and pancreas tissue were isolated from deceased donors under ethical approval from the Human Research Ethics Board of the University of Alberta (Pro00013094, Pro00001754) and obtained from the NIDDK-funded Integrated Islet Distribution Program (IIDP) (RRID: SCR _014387). All donors’ families gave informed consent for the use of pancreatic tissue in research and were not financially compensated.

### Mouse glucose tolerance tests

Animals were fasted for 5 h before intraperitoneal administration of 1.5 g kg^–1^ glucose. Specific antagonists were given 2 h before glucose at 1,500 nmol kg^–1^ doses, and tirzepatide was given 1 h before glucose, all by intraperitoneal injection. Glucose was measured with a handheld glucometer (Contour Blue, Bayer). Fasting glycemia was measured at time 0, 1 h following the administration of glucose. The AUC was calculated using the fasting glucose value for each animal. All wild-type mice were purchased from Jackson Laboratories (cat. no. 000664) between 8 and 12 weeks of age. All beta cell *Gipr*-knockout mice were bred in-house as previously described^10^. All mouse procedures were approved and performed in accordance with the Duke University Institutional Animal Care and Use Committee.

### Availability of materials

All reagents described within are available for distribution upon reasonable request to a corresponding author.

### Statistical tests

Sample sizes for mouse and cell experiments were based on power calculations using previously generated data with a similar experimental protocol. Human islet experiments were conducted based on equipment capabilities and experiment design. The appropriate statistical test was done and indicated in the chart below for each individual experiment. For ANOVA, Tukey post-hoc analyses were performed to identify specific differences. For all statistical tests, a *P* value of less than 0.05 was used to identify statistically different values. Specific statistical tests are indicated in Extended Data Table 3, and results are available for each data panel in the supplementary raw data files. All values presented are means ± s.e.m.

### Reporting summary

Further information on research design is available in the Nature Portfolio Reporting Summary linked to this article.

### Supplementary information

Reporting Summary

### Source data

Source Data Fig. 1 Excel workbook with raw data

Source Data Fig. 2 Excel workbook with raw data

Source Data Fig. 3 Excel workbook with raw data

Source Data Table 1 Excel workbook with raw data

Source Data Extended Data Fig. 1 Excel workbook with raw data

Source Data Extended Data Fig. 2 Excel workbook with raw data

Source Data Extended Data Fig. 3 Excel workbook with raw data

Source Data Extended Data Fig. 4 Excel workbook with raw data

Source Data Extended Data Table 1 Excel workbook with raw data

## Figures and Tables

**Extended Data Table 1:**
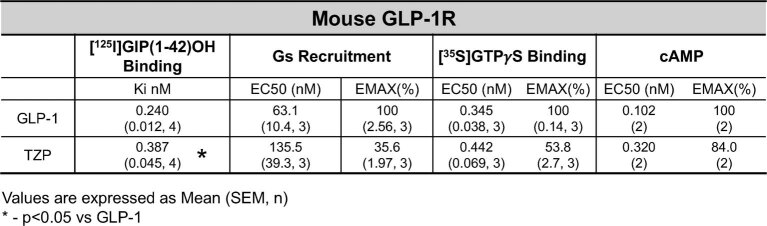
Mouse GLP-1R pharmacology

Receptor pharmacology of the mouse GLP-1R in response to either GLP-1 or tirzepatide (TZP). Values are expressed as mean (SEM, n). * - p < 0.05 vs GLP-1.

Source data

**Extended Data Table 2:**
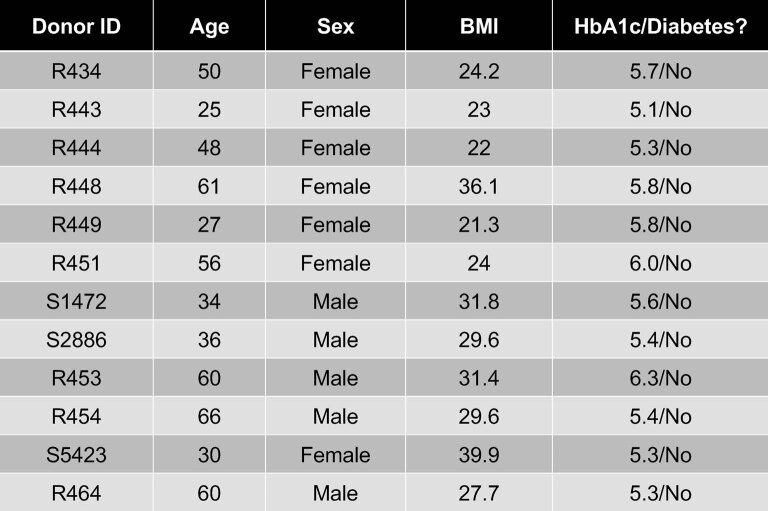
Human islet donor characteristics

Donor characteristics for human islets. BMI – body mass index. HbA1c – hemoglobin A1C.

**Extended Data Table 3:**
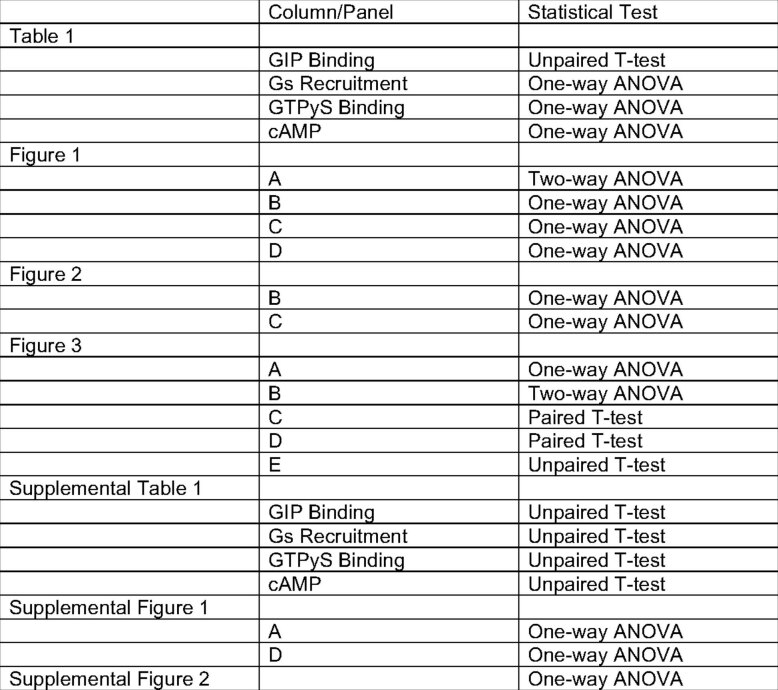
Statistical Tests used

The statistical test used for each data set is indicated by figure number and panel.

## Data Availability

All raw data have been provided to the journal and are available upon reasonable request to a corresponding author. Source data are provided with this paper.
