# Supplementary material for: The incretin co-agonist tirzepatide requires GIPR for hormone secretion from human islets
Source: Nat Metab. 2023 Jun 5;5(6):945–54. doi: 10.1038/s42255-023-00811-0 (PMC10290954; doi:10.1038/s42255-023-00811-0)
Supplement: Supplementary file 1 — Reporting Summary [file 42255_2023_811_MOESM1_ESM.pdf]

nature portfolio

Corresponding author(s): Kyle Sloop, Timo Muller, Jonathan Campbell

Last updated by author(s): March 28, 2023

Reporting Summary

Nature Portfolio wishes to improve the reproducibility of the work that we publish. This form provides structure for consistency and transparency in reporting. For further information on Nature Portfolio policies, see our Editorial Policies and the Editorial Policy Checklist.

Statistics

For all statistical analyses, confirm that the following items are present in the figure legend, table legend, main text, or Methods section.

n/a	Confirmed	
☐	☒	The exact sample size (*n*) for each experimental group/condition, given as a discrete number and unit of measurement
☐	☒	A statement on whether measurements were taken from distinct samples or whether the same sample was measured repeatedly
☐	☒	The statistical test(s) used AND whether they are one- or two-sided<br>*Only common tests should be described solely by name; describe more complex techniques in the Methods section.*
☒	☐	A description of all covariates tested
☐	☒	A description of any assumptions or corrections, such as tests of normality and adjustment for multiple comparisons
☐	☒	A full description of the statistical parameters including central tendency (e.g. means) or other basic estimates (e.g. regression coefficient) AND variation (e.g. standard deviation) or associated estimates of uncertainty (e.g. confidence intervals)
☐	☒	For null hypothesis testing, the test statistic (e.g. *F*, *t*, *r*) with confidence intervals, effect sizes, degrees of freedom and *P* value noted<br>*Give P values as exact values whenever suitable.*
☒	☐	For Bayesian analysis, information on the choice of priors and Markov chain Monte Carlo settings
☒	☐	For hierarchical and complex designs, identification of the appropriate level for tests and full reporting of outcomes
☒	☐	Estimates of effect sizes (e.g. Cohen's *d*, Pearson's *r*), indicating how they were calculated

Our web collection on statistics for biologists contains articles on many of the points above.

Software and code

Policy information about availability of computer code

Data collection	No software was used in the data collection
Data analysis	Data was analyzed with Graph Pad Prism ver 9.2.0.

For manuscripts utilizing custom algorithms or software that are central to the research but not yet described in published literature, software must be made available to editors and reviewers. We strongly encourage code deposition in a community repository (e.g. GitHub). See the Nature Portfolio guidelines for submitting code & software for further information.

Data

Policy information about availability of data

All manuscripts must include a data availability statement. This statement should provide the following information, where applicable:

- Accession codes, unique identifiers, or web links for publicly available datasets
- A description of any restrictions on data availability
- For clinical datasets or third party data, please ensure that the statement adheres to our policy

All raw data has been provided to the journal and is available upon reasonable request to a corresponding author.

Human research participants

Policy information about studies involving human research participants and Sex and Gender in Research.

Reporting on sex and gender	The available information on human islet donors is included in Supplemental Table 2.
Population characteristics	N/A
Recruitment	N/A
Ethics oversight	N/A

Note that full information on the approval of the study protocol must also be provided in the manuscript.

Field-specific reporting

Please select the one below that is the best fit for your research. If you are not sure, read the appropriate sections before making your selection.

☒ Life sciences ☐ Behavioural & social sciences ☐ Ecological, evolutionary & environmental sciences

For a reference copy of the document with all sections, see nature.com/documents/nr-reporting-summary-flat.pdf

Life sciences study design

All studies must disclose on these points even when the disclosure is negative.

Sample size	We used a sample size of at least 6/group for in vivo glucose tolerance testing, with is based on our previous experience with in vivo pharmacology in mice. For mouse islet perifusions, we used a minimum of 5/group collected over two independent experiments to ensure both proper statistical power and reproducibility across cohorts of mice and experimental days. For human islet perifusions, we used a minimum of 3/group for each individual islet donor. This is based on group sizes obtainable in a single experiment, donor islet availability. Summary data is provided across eight independent donors to provide quantification of donor variability and the reproducibility of our findings. For mouse studies (islet perifusion and glucose tolerance), power calculations were based on previous work in our group. No power calculations were performed for human islet perifusions, the experiments were based on capabilities and availability of donor material. The reproducibility of our data across individual donors indicated our sample sizes were sufficient.
Data exclusions	All raw data collected was used in the analysis and generation of final data figures.
Replication	Mouse islet perifusion studies were performed over two independent experimental days. All data collected was used in the final data sets Mouse in vivo studies were performed in a cross over manner over multiple days. All data collected was used in the final data sets. For human islet perifusions, reproducibility was assesses across multiple donors and provided as summary data. All data collected was used in the final data sets. For cell based experiments, each experimental data point was the average of three technical replications, performed in a single experiment. The group sizes represent the number of experiments.
Randomization	For mouse in vivo experiments, mice were randomly distributed to individual groups. Each group was received more than one treatment in a cross over manner. For islet experiments (both mouse and human), islets were randomly picked into individual batches of islets prior to experiment. For cell based assays, the individual treatment wells were randomly assigned.
Blinding	Due to the nature of these experiments, investigators were not blinded to the groups. For in vivo experiments, investigators were required to be aware of the reagents being administered to the mice. The primary output of blood glucose was collected and analyzed after the end of the experiment. For islet and cell based experiments, the investigator was required to be aware of the individual groups to ensure accurate execution of the experiment. The primary output of hormone concentrations are measured in a semi-automatic way and analysis after the end of the experiment. The investigator conducting the experiment differed from the investigator analyzing the data. All raw data collected was used in the analysis.

Reporting for specific materials, systems and methods

We require information from authors about some types of materials, experimental systems and methods used in many studies. Here, indicate whether each material, system or method listed is relevant to your study. If you are not sure if a list item applies to your research, read the appropriate section before selecting a response.

Materials & experimental systems

n/a	Involved in the study
☒	☐ Antibodies
☐	☒ Eukaryotic cell lines
☒	☐ Palaeontology and archaeology
☐	☒ Animals and other organisms
☒	☐ Clinical data
☒	☐ Dual use research of concern

Methods

n/a	Involved in the study
☒	☐ ChIP-seq
☒	☐ Flow cytometry
☒	☐ MRI-based neuroimaging

Eukaryotic cell lines

Policy information about cell lines and Sex and Gender in Research

Cell line source(s): HEK293T cells (ATCC, USA)

Authentication: Authentication according to the manufacturer's website:
The 293T cell line, originally referred as 293tsA1609neo, is a highly transfectable derivative of human embryonic kidney 293 cells, and contains the SV40 T-antigen.This cell line is competent to replicate vectors carrying the SV40 region of replication. It gives high titers when used to produce retroviruses. It has been widely used for retroviral production, gene expression and protein production. Product related references include DuBridge et al., Mol Cell Biol. 1987 Jan;7(1):379-87 and Pear et al., Proc Natl Acad Sci U S A. 1993 Sep 15;90(18):8392-6. https://www.lgcstandards-atcc.org/Products/All/CRL-3216.aspx?geo_country=de#generalinformation

Mycoplasma contamination: cell lines were free of mycoplasm contaminations

Commonly misidentified lines (See ICLAC register): no misidentified cell lines were used in the manuscript

Animals and other research organisms

Policy information about studies involving animals; ARRIVE guidelines recommended for reporting animal research, and Sex and Gender in Research

Laboratory animals: Figure 1A - In house bred mice on a C57BL/6J background. The controls are Gipr Flox/Flox mice and the knockouts are MIP-CreERT:GIpr Flox Flox mice. All mice are treated with tamoxifen at 8 weeks of age and islets were isolated at 12 weeks of age.
Figure 1B - C57BL/6 male mice with islets isolated at 12 weeks of age
Figure 1C - C57BL6J male mice, 14 weeks old
Extended Data Figure 1 - C57BL6J male mice, 14 weeks old
Extended Data Figure 1B - C57BL6J mice 14 weeks old
Extended Data Figure 1C - Glp1r knockout mice on a C57BL6J background. Control mice are wild-type littermates. Mice are 13 weeks old.
Extended data Figure 1D - C57BL6J male mice, 14 weeks old

All mice were housed in standard vivarium conditions - 21C, 40-60% humidity, 12:12 light:dark cycle.

Wild animals: no wild animals were used in the study

Reporting on sex: All mice were male. The sex of human islet donors is indicated in Extended Data Table 2

Field-collected samples: no field collected animals were used in the study

Ethics oversight: All mouse procedures were approved and performed in accordance with the Duke University Institutional Animal Care and Use Committee.

Note that full information on the approval of the study protocol must also be provided in the manuscript.
